# Learning dynamic treatment strategies for coronary heart diseases by artificial intelligence: real-world data-driven study

**DOI:** 10.1186/s12911-022-01774-0

**Published:** 2022-02-15

**Authors:** Haihong Guo, Jiao Li, Hongyan Liu, Jun He

**Affiliations:** 1 School of Information, Renmin University of China, 59 Zhongguancun Street, Haidian District, Beijing, 100872 China; 2 Institute of Medical Information/Medical Library, Chinese Academy of Medical Sciences and Peking Union Medical College, Beijing, China; 3 Key Laboratory of Data Engineering and Knowledge Engineering, Ministry of Education, Beijing, China; 4 School of Economics and Management, Tsinghua University, Beijing, China

**Keywords:** Dynamic treatment strategies, Coronary heart diseases, Artificial intelligence, Supervised reinforcement learning, Deep sequential recommendation

## Abstract

**Background:**

Coronary heart disease (CHD) has become the leading cause of death and one of the most serious epidemic diseases worldwide. CHD is characterized by urgency, danger and severity, and dynamic treatment strategies for CHD patients are needed. We aimed to build and validate an AI model for dynamic treatment recommendations for CHD patients with the goal of improving patient outcomes and learning best practices from clinicians to help clinical decision support for treating CHD patients.

**Methods:**

We formed the treatment strategy as a sequential decision problem, and applied an AI supervised reinforcement learning-long short-term memory (SRL-LSTM) framework that combined supervised learning (SL) and reinforcement learning (RL) with an LSTM network to track patients’ states to learn a recommendation model that took a patient’s diagnosis and evolving health status as input and provided a treatment recommendation in the form of whether to take specific drugs. The experiments were conducted by leveraging a real-world intensive care unit (ICU) database with 13,762 admitted patients diagnosed with CHD. We compared the performance of the applied SRL-LSTM model and several state-of-the-art SL and RL models in reducing the estimated in-hospital mortality and the Jaccard similarity with clinicians’ decisions. We used a random forest algorithm to calculate the feature importance of both the clinician policy and the AI policy to illustrate the interpretability of the AI model.

**Results:**

Our experimental study demonstrated that the AI model could help reduce the estimated in-hospital mortality through its RL function and learn the best practice from clinicians through its SL function. The similarity between the clinician policy and the AI policy regarding the surviving patients was high, while for the expired patients, it was much lower. The dynamic treatment strategies made by the AI model were clinically interpretable and relied on sensible clinical features extracted according to monitoring indexes and risk factors for CHD patients.

**Conclusions:**

We proposed a pipeline for constructing an AI model to learn dynamic treatment strategies for CHD patients that could improve patient outcomes and mimic the best practices of clinicians. And a lot of further studies and efforts are needed to make it practical.

**Supplementary Information:**

The online version contains supplementary material available at 10.1186/s12911-022-01774-0.

## Background

Coronary heart disease (CHD) has become the leading cause of death and one of the most serious epidemic diseases worldwide [1]. It is estimated that 126.5 million people worldwide have CHD, that 8.9 million people died of CHD per year [1], and that 18.2 million American adults have CHD and 363, 452 died from CHD in 2016 [2]. Eleven million Chinese residents had CHD, and the mortality rate was 120.18 per 100 thousand in 2017 [3]. CHD is characterized by urgency, danger and severity; thus, personalized dynamic treatment in the ICU is particularly important [4]. A series of general guidelines on rational drug use for CHD have been made by experts [5–7]. However, one ideal treatment strategy may be effective for some patients but not for others, and even the same patient might need different treatment strategies during different stages of the CHD process. Additional file 2: Figure S1 shows an example of the dynamic treatment strategies administrated to a CHD patient.

Early studies of dynamic treatment strategies were conducted mainly on simulation and clinical trial datasets that were limited in their reflection of real-world situations [8–10]. With the increasing availability of electronic health records (EHRs), leveraging massive real-world EHRs to improve treatment strategies has become an ad hoc research direction [11]. Some studies have been conducted on treatment recommendations for multimorbidity [12–17], while others have focused on specific diseases, including sepsis [18–20], oncology [21], non-small-cell lung cancer [8, 9], breast cancer [22–24], cerebral infarction disease [25], diabetes [26, 27], hypertension [28], hypercholesterolemia [29], AIDS [30], adolescent depression [31–34], bipolar disorder [35, 36], anxiety disorders [37], paediatric generalized schizophrenia [38], graft versus host disease [39], thrombosis [40], and paediatric cystic fibrosis [41]. Several works have modelled personalized treatment pathways [42, 43], built automatic clinical guidelines [44–46], and developed optimized exercise prescription systems [47] for cardiovascular diseases. Few works have been conducted on intelligent learning of dynamic treatment strategies for CHD [48], especially dynamic drug recommendations according to the evolving health status of CHD patients.

The methods used to design artificial intelligence (AI) to identify dynamic treatment strategies can be classified into three main categories: (1) rule-based expert systems, which map diseases to treatments based on heuristic rules [44]; (2) supervised learning (SL) methods, which generate treatment recommendations by utilizing the similarity of patients or match diseases with treatments via classification, including pattern-based methods and deep learning [12, 49], and more recently attention and memory-augmented network (AMANet) [50]; and (3) reinforcement learning (RL) methods [51, 52], which address delayed rewards and infer an optimal strategy based on non-optimal treatment behaviours, including value-based RL [18–20] and direct policy optimization [53]. Other methods include outcome weighted learning [54], augmentation and relaxation learning [55], and ensemble machine learning [56]. Each kind of method has respective advantages and drawbacks. Taking SL methods as an example, on the one hand, they are adept at mining the experience of doctors from labelled data; on the other hand, their prerequisite of assuming that the treatment label provided by doctors is optimal is not always the case [57], so they may learn some wrong things. RL methods infer an optimal treatment strategy according to the delayed reward set up mainly by a patient outcome, but they may recommend treatments that are obviously different from a doctor’s prescription due to the lack of supervision, which may cause high risk in clinical practice [58]. These two kinds of methods can supplement each other by combining an evaluation signal and an indicator signal to learn an integrated treatment policy [14, 15].

We aimed to build and validate an AI model for dynamic treatment recommendations for CHD patients with the goal of improving patient outcomes and learning the best practices of clinicians to help clinical decision support in treating CHD patients. Inspired by Wang et al. [15] and the aforementioned studies, we applied an AI model of supervised reinforcement learning-long short-term memory (SRL-LSTM) to learn dynamic treatment strategies for CHD patients by using real-world EHRs and compared it with several state-of-the-art SL models and RL models. We used a random forest algorithm to calculate the feature importance of both the clinician policy and the AI policy to illustrate the interpretability of the AI model. Case studies were conducted to analyse the similarities and differences between the AI-recommended treatment actions and the clinicians’ actual treatment decisions.

**Fig. 1:**
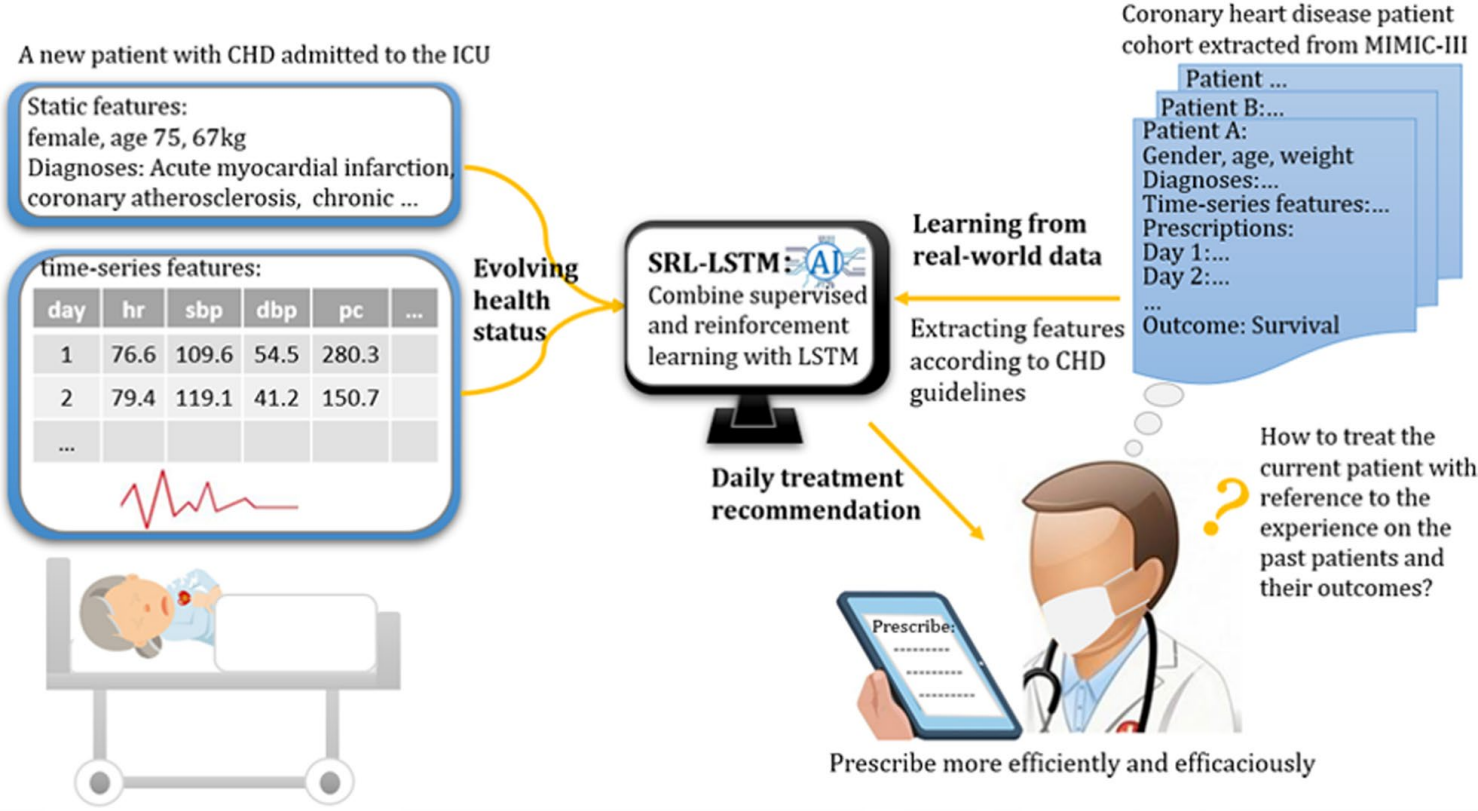
Framework of the study design. An AI model SRL-LSTM is learned from historical CHD cohort. For a patient with CHD admitted to the ICU, the model takes the patient’s static and time-series features as input and provides a daily treatment recommendation

Figure 1 shows the framework of our study. To help clinicians prescribe efficiently and efficaciously to treat patients admitted to ICU with CHD, as shown in Fig. 1, we first train an AI model SRL-LSTM based on historical CHD cohort extracted from MIMIC-III V1.4, an open access and anonymized real-world ICU database. The model combines SL and RL with an LSTM network to track the patients’ states and learns dynamic treatment policies. When a patient with CHD is admitted to the ICU, we feed the patient’s evolving health status to the model, including diagnoses, demographics and time-series variables of the patient till the current day extracted according to CHD guidelines. The model can then provide us a daily treatment recommendation in the form of whether to take specific drugs.

## Material and methods

### Overall approach and cohort

We formed the treatment strategy as a sequential decision problem and applied an AI SRL-LSTM framework that combined SL and RL with an LSTM network to track patients’ states to learn a recommendation model that took the patient’s diagnosis and evolving health status as input and gave a daily treatment recommendation in the form of whether to take specific drugs. The outcome of interest was the hospital mortality of the selected cohort.

**Fig. 2:**
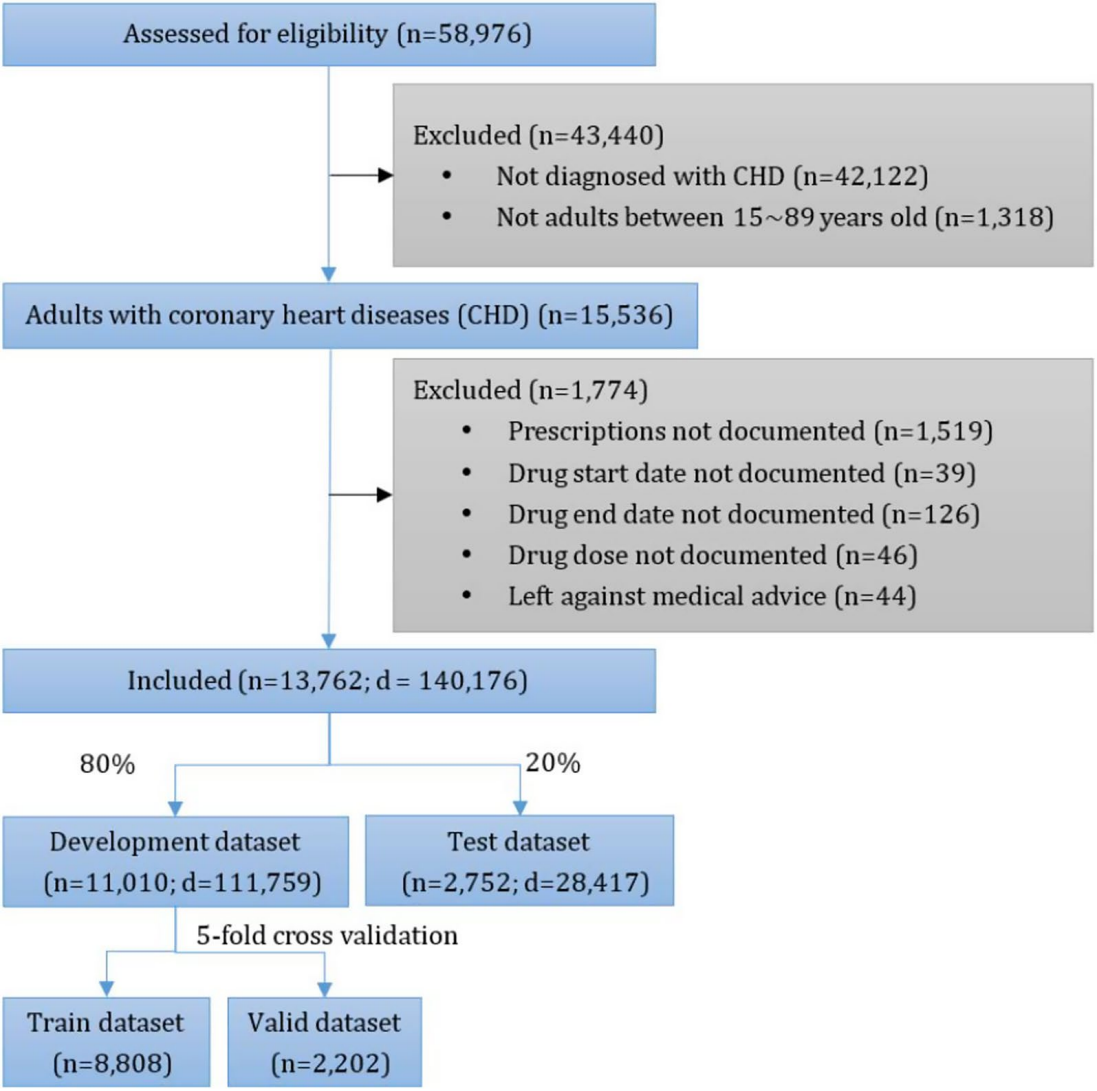
Patient admission inclusion diagram. n is the number of hospital admissions, and d is the hospitalization days

The cohort was selected from MIMIC-III V1.4, an open access and anonymized real-world ICU database containing 58,976 admissions from 2001 to 2012 in 5 ICUs of a teaching hospital in the Northeast United States [59–61]. We included adult patients diagnosed with CHD. CHD is a class of heart diseases caused by myocardial ischaemia, hypoxia, or necrosis because of narrowing or occlusion of the lumen as a result of coronary atherosclerosis [7], which is also called ischaemic heart disease [62]. Following [2], we used the International Classification of Diseases (ICD), 9th edition revision codes 410 to 414 [63] to indicate CHD. The patient admission inclusion diagram is shown in Fig. 2.

### Data extraction and preprocessing

We extracted the prescriptions of the CHD cohort, which contained 1,292,650 records and 2477 drugs. To ensure statistical significance, we selected the top 500 drugs that covered 98.0% of the prescriptions. The prescription action was coded as one-hot for each day. If the prescription of a patient contained drugs out of the selected drug list on one day, then the patient was still included and only the drugs within the list were scanned and coded. The data were included from hospital admission to discharge, resulting in a total of 140,176 action days.

Each patient had 1 to 39 diagnoses coded by ICD-9, and a total of 3719 diseases were involved. All the diagnoses of a CHD patient were used as the input layer, and each of them was embedded with 40 hidden nodes regarding to the maximum number of diagnoses observed. The embedding was conducted as follows. First, all ICD-9 codes used to identify the CHD cohort were sorted in frequency descending order; then, the ICD-9 codes of each CHD patient were replaced by their indexes in descending order, and zeros were padded to make the length of each patient’s diagnosis sequence equal to that of the largest diagnoses sequence. The model can be adjusted to deal with longer or shorter sequence and easily retrained on new dataset containing new patients with more or less diagnoses. Finally, the embedding layer in Keras [64] was applied to turn the positive integers (indexes) into dense vectors of fixed size. We analysed the rationality of the embedding method in both development set and test set. For both the development set and test set, we first calculated the Euclidean distances between each two patients in both the original index space and the embedding space respectively. Then we divided the distances in the original index space into 3 equal groups: the closer group, the middle-distance group, and the distant group. We calculated the mean distances of each group in the original index space and the mean distances of corresponding patient pairs in each group in the embedding space. Additional file 1: Table S1 shows that, for both development set and test set, the closer groups in the original index spaces have closer mean distances in the embedding spaces, the distant groups in the original index spaces have greater mean distances in the embedding spaces. And the mean distances of corresponding groups in the development set and test set are close. This demonstrated that the embedding method could rationally keep the relative distances between patients in terms of their diagnoses.

We identified monitoring indicators and risk factors for the CHD patients by searching CHD related guidelines [5–7], handbooks [4], reports [3], and papers [1, 2]. Then, for each hospital admission, we extracted static variables and time-series variables that were recorded for at least 20% of the sampled hospital admissions. Finally, the model features included diagnoses, demographics, electrocardiogram and haemodynamic monitoring results, vital signs, ventilation parameters, lab values, and output events. Among them, demographics such as gender, age, and weight are the basic risk factors for the CHD patients, and electrocardiogram monitoring of heart rhythm and heart rate are basic monitoring items for the CHD patients for detecting each kind of arrhythmia and the situation of myocardial ischaemia. Haemodynamic instability is a prominent manifestation in patients with severe cardiovascular disease. The monitoring of haemodynamic indexes is particularly important in the condition evaluation and rescue treatment of patients with severe cardiovascular disease. Systolic, diastolic, and mean blood pressure, systolic, diastolic, and mean pulmonary artery pressure (PAP), central venous pressure, shock index, cardiac index, and systemic vascular resistance index (SVRI) are important haemodynamic indexes. Other vital signs, such as temperature, respiratory rate, SpO_2_, and Glasgow coma scale (GCS) score, are important indexes for severity grading in the ICU and are also suitable for CHD patients in the ICU. FiO_2_ and mechanical ventilation are important indicators for admission to the ICU and for judging the prognosis of the CHD patients. Both hyperglycaemia and hypoglycaemia are important cardiovascular risk factors; thus, blood glucose values were included. Lactate dehydrogenase, creatine kinase (CK), CK-MB isoenzyme, and troponin T are markers of myocardial injury and play important roles in clinical diagnosis, condition monitoring and risk stratification of acute myocardial infarction and other diseases associated with myocardial injury. Renal insufficiency is a common and important complication in patients with severe cardiovascular disease and is one of the predictors of poor prognosis; thus, we included indexes such as creatinine, blood urea nitrogen, and daily urine output, which could reflect renal injury. Heart disease is often associated with liver insufficiency, so indexes that could reflect liver function were included, such as alkaline phosphatase, serum glutamic-oxaloacetic transaminase (SGOT), serum glutamic pyruvic transaminase (SGPT), total bilirubin, albumin, partial thromboplastin time (PTT), prothrombin time (PT), and international normalized ratio (INR). Partial pressure of oxygen (PaO2) was included to reflect hypoxia. Other basic laboratory values were also used, including routine blood indexes such as haemoglobin, white blood cell count, and platelet count; electrolyte indexes such as potassium, sodium, magnesium, calcium, ionized calcium, and chloride; and acid base balance indexes including pH, carbon dioxide (CO2), PaCO2, base excess, bicarbonate, and lactate.

Variable heart rhythms were divided into 25 sub-types, including atrial fibrillation, atrial flutter, A paced, V paced, AV paced, left bundle branch block, right bundle branch block, sinus arrhythmia, sinus bradycardia, sinus rhythm, sinus tachycardia, supra ventricular tachycardia, ventricular tachycardia, ventricular fibrillation, multifocal atrial tachycardia, paroxysmal atrial tachycardia, wandering atrial pacemaker, first degree AV block, second degree AV block Wenckebach—Mobitz1, second degree AV block—Mobitz 2, complete heart block, junctional rhythm, idioventricular, asystole, and others, which were coded as one-hot sub-variables. We divided the time-series data of each hospital admission into different units, which were set to 24 h following [15] since it was the minimum interval of prescription in MIMIC-III. Following [15, 18], variables with multiple data points in one unit were averaged (for instance, systolic blood pressure) or summed (for instance, urine output).

The quality of the data was improved in the preprocessing step. Variables with different measurement units were unified. For example, pound weights were converted to kilograms, and temperatures in Fahrenheit were converted to temperatures in Celsius. Variables extracted from different tables, such as *labevents* and *chartevents* in MIMIC-III, were combined, and duplicates were dropped according to the keys of hospital admission ID and chart time. Several composite variables were calculated by their composing sub-variables. For instance, some of the GCS values were summed by their sub-variables: GCS eye, GCS verbal, and GCS motor; and the shock index was calculated by heart rate dividing systolic blood pressure. Because pulse was not available in MIMIC-III, it was replaced by heart rate according to [18]. We detected the outliers with a frequency histogram and normal probability graph and removed them to cap all the variables to clinically plausible values. Variables not normally distributed were transformed to their logarithms as appropriate, all the variables were normalized, and the missing variables were imputed by k-nearest neighbours (KNN).

### AI models

#### Model preliminaries

In this paper, the dynamic treatment strategy was modelled as a partially observed Markov decision process with finite time steps. Let ${D=\left\{\left({S}_{i,t}, {A}_{i,t}, {S}_{i,t+1}, {r}_{i,t} \right):t=1,\dots ,{T}_{i}\right\}}_{i=1}^{n}$ denote the observed dataset, where $n$ is the number of patient admission trajectories. For each patient admission trajectory$i$, ${T}_{i}$ is the total hospitalization days; $\left({S}_{i,t}, {A}_{i,t}, {S}_{i,t+1}, {r}_{i,t}\right)$ shows the transitions from the $t$ th day to the $(t+1)$ th day, where ${S}_{i,t}$ is the current observed state; ${A}_{i,t}$=(${a}_{i,t}^{1}$,${a}_{i,t}^{2}$, …,${a}_{i,t}^{k},\dots ,{a}_{i,t}^{K}$) is the actual medications prescribed by clinicians, where $K=500$ and ${a}_{i,t}^{k}\in \left\{\mathrm{0,1}\right\}$ represents whether or not to take drug$k$; ${S}_{i,t+1}$ is the state on the next day after taking action${A}_{i,t}$, ${S}_{i,{T}_{i}+1}=0$ denotes the termination of the trajectory, and ${r}_{i,t}$ is the reward gained. Given the current observed state${S}_{i,t}$, our goal was to learn a policy $\mu ({S}_{i,t}|{\theta }^{\mu })$ to select an action (drug combinations) $\widehat{{A}_{i,t}}$ by maximizing the expected return and minimizing the difference from clinicians’ decision${A}_{i,t}$, where $\theta$ refers to the parameters within the respective network. A critic network $Q(S,A|{\theta }^{Q})$ was built to estimate the expected return, which was the accumulated discount reward from this state to the end of the trajectory [65].

#### SRL-LSTM

We applied an AI SRL-LSTM framework [15] to minimize the following objective loss function:

1 $$L\left({\theta }^{\mu }\right)= \varepsilon *{L}_{RL}\left({\theta }^{\mu }\right)+\left(1-\varepsilon \right)*{L}_{SL}\left({\theta }^{\mu }\right),$$

where ${L}_{RL}\left({\theta }^{\mu }\right)$ is the loss for RL, which aims at minimizing the negative expected return to maximize the positive expected return; ${L}_{SL}\left({\theta }^{\mu }\right)$ is the loss for SL, which aims at minimizing the difference between the recommended action and the real action made by the clinicians; and $\varepsilon$ is a weight parameter to trade off the objective between them.

We used a deep deterministic policy gradient (DDPG) for the RL part and cross entropy as the supervisor. In DDPG [66], there are two agents, actor $\mu (S|{\theta }^{\mu })$ and critic $Q(S,A|{\theta }^{Q})$, each of which has a target network ${\mu }^{^{\prime}}(S|{\theta }^{{\mu }^{^{\prime}}})$ and ${Q}^{^{\prime}}(S,A|{\theta }^{{Q}^{^{\prime}}})$ with the same structure but different parameter update frequencies to ensure the robustness of the model. LSTM with a time window of 5 days was adopted to track the historical observed states within the actor and critic networks. Their network architectures are shown in Additional file 3: Figure S2, and the parameters were updated by the Adam optimizer.

For a random minibatch of $N$ transitions $\left({S}_{i,t}, {A}_{i,t}, {S}_{i,t+1}, {r}_{i,t}\right)$ from $D$, the critic $Q\left(S,A|{\theta }^{Q}\right)$ can be updated by minimizing the mean square error (MSE) loss:

2 $${L}_{Q}\left({\theta }^{Q}\right)=\frac{1}{N} {\sum }_{i=1}^{N}{({y}_{i}-Q\left({S}_{i,t}, {A}_{i,t}|{\theta }^{Q}\right))}^{2},$$

where ${y}_{i}= {r}_{i,t}+ \gamma {Q}^{\mathrm{^{\prime}}}({S}_{i,t+1},{\mu }^{\mathrm{^{\prime}}}({S}_{i,t+1}|{\theta }^{{\mu }^{\mathrm{^{\prime}}}})|{\theta }^{{Q}^{\mathrm{^{\prime}}}})$, and $\gamma$ is the discount ratio of reward. The RL loss of the actor $\mu (S|{\theta }^{\mu })$ is defined as:

3 $$\widehat{{A}_{i,t}}= \mu \left({S}_{i,t}|{\theta }^{\mu }\right)=\left(\widehat{{a}_{i,t}^{1}}, \widehat{{a}_{i,t}^{2}},\dots ,\widehat{{a}_{i,t}^{K}}\right), K=500$$

4 $${L}_{RL}\left({\theta }^{\mu }\right)=-{\mathbb{E}}\left[Q\left(S,\widehat{A} |{\theta }^{Q}\right)\right] \approx -\frac{1}{N} {\sum }_{i=1}^{N}\left(Q\left({S}_{i,t}, \widehat{{A}_{i,t}}|{\theta }^{Q}\right)\right),$$

The SL loss of the actor is defined as:

5 $${L}_{SL}\left({\theta }^{\mu }\right)=-\frac{1}{N} {\sum }_{i=1}^{N}{\sum }_{k=1}^{K}{a}_{i,t}^{k}\mathrm{log}\left(\widehat{{a}_{i,t}^{k}}\right),$$

Then, the target networks are updated by:

6 $${\theta }^{{Q}^{\mathrm{^{\prime}}}}= \tau {\theta }^{Q}+\left(1-\tau \right){\theta }^{{Q}^{\mathrm{^{\prime}}}},$$

7 $${\theta }^{{\mu }^{\mathrm{^{\prime}}}}= \tau {\theta }^{\mu }+\left(1-\tau \right){\theta }^{{\mu }^{\mathrm{^{\prime}}}},$$

where $\tau$ is a weight to control the updating ranges of the parameters.

#### Models for comparison

We compared the SRL-LSTM model with the following state-of-the-art models that were suitable for learning daily medication recommendation strategies:

- Dual-LSTM: Dual-LSTM [50] took the treatment recommendation as a classification problem, which encodes disease information and time-series variables using LSTM respectively and then concatenates the two encoding vectors for the final classification layer.
- AMANet: AMANet is a classification model for dual-view sequential learning based on attention and memory mechanisms [50]. The original AMANet predicted medications for each patient visit based on ordered diagnoses and medical procedures by treating the diagnoses and procedures in the current visit as two sequential views. Our purpose was to predict the daily medications based on diagnoses and daily time-series variables, so we replaced the token embedding of procedures in the original AMANet with an LSTM layer for the time-series variables.
- Direct policy optimization (DPO) with LSTM: DPO is a RL method to directly learn a policy without learning an extra model of treatment effectiveness [53]. We used the same architecture of the actor network and reward *r* as defined in the SRL-LSTM model and learned a single model that directly predicts which treatment is optimal by optimizing a surrogate loss:
  $${L}_{DPO}=-\frac{1}{{T}_{i}} {\sum }_{t=1}^{{T}_{i}}{\sum }_{k=1}^{K}{a}_{i,t}^{k}\mathrm{log}\left(\widehat{{a}_{i,t}^{k}}\right)*\frac{\left({V}_{i,t}-\overline{V }\right)}{S\left(V\right)},$$
  where $${V}_{i,t}={\gamma }^{{T}_{i}-t}{r}_{i,{T}_{i}}$$, $$\overline{V }=\frac{1}{{\sum }_{i=1}^{N}{T}_{i}}{\sum }_{i=1}^{N}{\sum }_{t=1}^{{T}_{i}}{V}_{i,t}$$, and $$S\left(V\right)=\sqrt{\frac{{\sum }_{i=1}^{N}{\sum }_{t=1}^{{T}_{i}}{({V}_{i,t}-\overline{V })}^{2}}{{\sum }_{i=1}^{N}{T}_{i}-1}}$$.
- SRL-Multimorbidity: SRL-Multimorbidity [15] was developed for dynamic medication recommendations for multimorbidity with a combination of SL and RL. Its time-series features only include diastolic blood pressure, fraction of inspiration O2, Glasgow coma scale score, blood glucose values, systolic blood pressure, heart rate, pH, respiratory rate, blood oxygen saturation, body temperature, and urine output.

### Experiment setup

We randomly split the CHD dataset into a development set (80%) and a test set (20%) and applied 5-fold cross validation to the development set to investigate the balance of SL and RL. We trained the comparison models on the development set and compared them with the SRL-LSTM model on the test set. We trained the models through the TensorFlow (version 2.1.0) framework in Python (version 3.7.3) on a GPU- supported machine. The training process of the SRL-LSTM model is shown in Additional file 3: Figure S2 and described in the text in Additional file 1, and the hyperparameters are summarized in Additional file 1: Table S2.

### Evaluation

We adopted the estimated in-hospital mortality rates on both state-wise and trajectory-wise (or admission-wise) to measure whether the AI strategy model could reduce patient mortality. The estimated mortality rate is a universal metric for computational testing of treatment recommendation models when only retrospective data are available [15, 18–20]. The state-wise estimated in-hospital mortality rate was computed as follows. Step 1: We obtained the recommended actions for each patient state by the actor evaluation network and the expected returns of both the clinicians’ actual actions and the AI model recommended actions by the critic evaluation network. Step 2: We assigned the in-hospital mortality flag for all the expected returns of actual actions. Step 3: We discretized all the expected returns of actual actions into different units according to their distribution with 5% in each unit. Step 4: We calculated the average estimated mortality rate for each unit by the bootstrapping with 2000 re-samplings. Step 5: We discretized all the expected returns of recommended actions into each unit, and calculated the expected mortality number in each unit according to the number of states and the average mortality rate. Step 6: We calculated the state-wise expected in-hospital mortality rate by using the total expected mortality number to divide the total states.

The state-wise estimated in-hospital mortality rate took each patient state to represent a patient admission to make full use of the data; however, it was not the direct estimated in-hospital mortality rate. Therefore, we also calculated the trajectory-wise (admission-wise) estimated in-hospital mortality rate according to the above steps, with the expected returns of each state-action pair replaced by that of the initial state-action pair in each trajectory.

We used the mean Jaccard coefficient to measure the degree of consistency between prescription actions taken by the clinicians and those recommended by the AI model since the task belongs to multilabel classification [14–17]. The mean Jaccard is defined as follows:

8 $$\mathrm{J}= \frac{1}{M}{\sum }_{i=1}^{M}\frac{1}{{T}_{i}}{\sum }_{t=1}^{{T}_{i}}\frac{\left|{A}_{i,t}\cap \widehat{{A}_{i,t}}\right|}{\left|{A}_{i,t}\cup \widehat{{A}_{i,t}}\right|},$$

where $M$ is the number of patient admissions in the valid/test set. $J\in \left[\mathrm{0,1}\right]$, where $J=1$ indicates that the daily treatment actions recommended by the AI policy are exactly the same as those proposed by the clinicians; in contrast, $J=0$ indicates that none of the drugs recommended by the AI policy are the same as the drugs proposed by the clinicians for each day.

We analysed how the observed in-hospital mortality rate varies with the difference in treatment actions between the AI policy and the clinician policy. The treatment difference for the *i*th patient on the *t*th hospitalization day was defined as:

9 $${B}_{i,t}= {\sum }_{k=1}^{K}\left|{a}_{i,t}^{k}- \widehat{{a}_{i,t}^{k}}\right|,$$

Furthermore, a case study was conducted to see the similarities and differences between the AI-recommend treatment actions and the clinicians’ actual treatment decisions on both the surviving and expired patients.

### Interpretability analysis

We used a random forest model to estimate the importance of the features in decision making for the AI model and the clinicians [18] to gain some insight into the model representations and interpretability. The random forest model was fitted by all the test data over all the treatment periods. The independent variables of the model are patients’ feature (except diagnosis). The dependent variable is the real prescription action taken by the clinicians when calculating feature importance for the clinician policy, and the recommended action generated by the AI model of SRL-LSTM when calculating feature importance for the AI policy. We calculated the importance of the heart rhythm by accumulating the importance of its 25 sub-types.

## Results

### Distribution of the feature variables

Following the cohort selection and exclusion criteria, we included 13,762 admitted patients diagnosed with CHD. A detailed description of the cohort is provided in Additional file 1: Table S3. Table 1 shows the distribution of model features before normalization and imputation by KNN. In all, 65.6% of the CHD patients were male. CHD is a disease of old age, and 79.8% of the patients were over 60-years of age. A total of 29.1% suffered severe coma, and 38.0% suffered moderate coma during the hospital stay. The troponin T, CK and CK-MB isoenzyme levels were higher than the normal values because the patients’ myocardia were infarcted by the ischaemia hypoxia. The average values of blood glucose were higher than those in the common population, since four out of five patients had endocrine, nutritional, metabolic, and immune diseases. More than half of the CHD patients suffered kidney damage according to the creatinine and blood urea nitrogen values.

**Table 1:** Distribution of the feature variables of the CHD cohort

Items	Distribution	Items	Distribution
**Diagnoses**	3719, embedded with 40 hidden nodes	**Lab values (33, Mean, SD)**	
		Blood glucose	132.95 (46.76)
		Creatinine	1.60 (1.51)
**Demographics (3)**		Blood urea nitrogen	31.08 (22.16)
Male gender (N, %)	9,024 (65.6%)	Potassium	4.16 (0.52)
Age, years (Mean, SD)	69.87 (11.80)	Sodium	138.35 (4.21)
Weight (Mean, SD)	83.83 (20.91)	Magnesium	2.09 (0.32)
**Electrocardiogram monitoring results (2)**		Calcium	8.54 (2.32)
Heart rhythm	25 sub-types, binary coded	Ionized calcium	1.14 (0.14)
		Chloride	103.25 (5.56)
Heart rate (Mean, SD)	83.76 (14.31)	Carbon dioxide	26.37 (5.27)
**Haemodynamic monitoring (10, Mean, SD)**		Troponin T	1.08 (2.37)
Systolic blood pressure	118.16 (17.30)	Creatine kinase (CK)	448.85 (969.29)
Diastolic blood pressure	57.85 (10.22)	CK-MB isoenzyme	27.09 (57.04)
Mean blood pressure	76.13 (10.98)	Lactate dehydrogenase	408.74 (428.56)
Systolic PAP	38.37 (11.05)	Alkaline phosphatase	138.89 (151.13)
Diastolic PAP	19.34 (5.68)	SGOT	137.12 (512.22)
Mean PAP	29.45 (19.57)	SGPT	118.87 (402.44)
Central venous pressure	14.39 (18.73)	SGOT/SGPT ratio	1.15
Shock index	0.73 (0.18)	Total bilirubin	1.87 (3.90)
Cardiac index	2.77 (0.62)	Albumin	3.09 (0.81)
SVRI	1462.95 (380.42)	Haemoglobin	10.42 (1.68)
**Other vital signs (4, Mean, SD)**		White blood cells count	10.94 (6.20)
Temperature	36.83 (0.64)	Platelet count	236.24 (122.62)
Respiratory rate	19.72 (4.26)	PTT	42.73 (21.94)
SpO_2_	96.51 (3.03)	PT	16.47 (6.69)
GCS	12.22 (3.44)	INR	1.56 (0.88)
**Output events (1, mean, SD)**		pH	7.39 (0.08)
Daily urine output	1672.33 (1215.37)	PaO2	131.71 (70.42)
**Ventilation parameters (2)**		PaCO2	41.60 (8.68)
FiO2 (Mean, SD)	24.84 (11.72)	Base excess	0.51 (4.47)
Mechanical ventilation	Binary, if the value = 1 in the source tables or the FiO2 > 21, then it was set to be 1;otherwise, it was set to be 0	Bicarbonate	25.98 (4.47)
		Lactate	2.06 (1.73)
		PaO2/FiO2 ratio	498.87 (311.30)

### Performance of the AI model

**Fig. 3:**
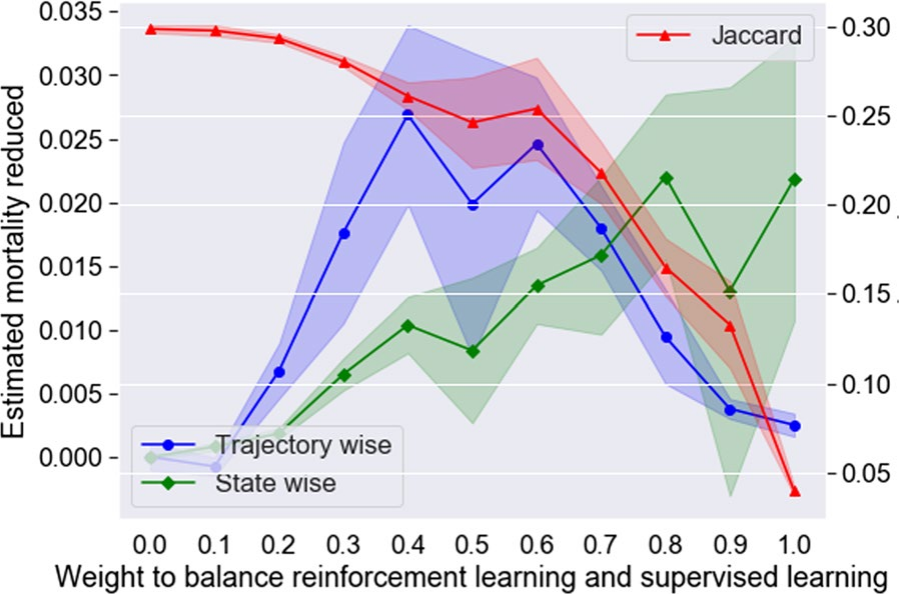
Performance of models with different weights to balance reinforcement learning and supervised learning in 5-fold cross validation. The dark lines in the middle indicate the mean values, and the shaded areas indicate 1 standard deviation above and below the mean values

Figure 3 presents the performance of the models with different weights to balance RL and SL in 5-fold cross validation. The SL-LSTM model ($\varepsilon =0.0, 100\% SL$) achieved the highest Jaccard value, indicating that it had the best ability to learn the experience of the clinicians; however, it did not show any ability to improve the outcome of the CHD patients on either the state-wise or trajectory-wise estimated in-hospital mortality rate. The RL-LSTM model ($\varepsilon =1.0, 100\% RL$) seemed to have the ability to substantially reduce the state-wise estimated in-hospital mortality rate; however, it had the lowest Jaccard value, indicating that its recommendation was quite different from the clinicians’ decision. The SRL-LSTM model with weight parameter $\varepsilon =0.4$ exhibited a relatively high performance in reducing both the trajectory-wise and state-wise estimated in-hospital mortality rates, while its Jaccard value was not greatly harmed by RL, and all three evaluation indicators had relatively small variation. Therefore, the AI model with 40% RL and 60% SL was preferable.

The proposed SRL-LSTM model ($\varepsilon =0.4, 40\% RL, 60\% SL$) was then tested against several recent methods, and was found to outperform them all (Table 2). The test dataset contained 2,752 patient admissions (trajectories) with 28,417 hospitalization days (states). A total of 9.56% of the patients died in the hospital under the clinicians’ actual treatment policy, and the state-wise mortality rate was 9.59%. The preferred AI model of SRL-LSTM could help reduce the trajectory-wise and state-wise estimated in-hospital mortality rate by 3.13% and 0.81% respectively, while keeping the Jaccard similarity (0.3110) close to the SL-LSTM model (0.3432), and its average recommended drug amount (27) was close to that of the clinicians. Considering that diagnoses codes might not be available at bedside, we conducted an ablation experiment by removing the disease information out from the SRL-LSTM model, and the result showed that it would slightly harm the performance by increasing the trajectory-wise estimated mortality rate by 0.98% and decreasing the Jaccard similarity score by 8.77%.

**Table 2:** Performance comparison on the test dataset

Method	Estimated mortality		Jaccard
	Trajectory-wise	State-wise	
Clinician’s policy	0.0956	0.0959	–
Dual-LSTM	0.0887	0.0935	0.3171
AMANet	0.0827	0.0895	0.3250
DPO-LSTM	0.0919	0.0930	0.2069
SRL-Multimorbidity	0.0948	0.0953	0.2610
SL-LSTM ($\varepsilon =0$)	0.0956	0.0967	0.3432
RL-LSTM ($\varepsilon =1.0$)	0.0752	0.0721	0.0342
**SRL-LSTM (**${\varvec{\varepsilon}}=0.4$**)**	**0.0643**	**0.0878**	**0.3110**
SRL-LSTM($\varepsilon =0.4$, w/o diagnosis codes)	0.0741	0.086	0.2233

The bold indicates the prefered AI model to learn dynamic treatment strategies for CHD patients

**Fig. 4:**
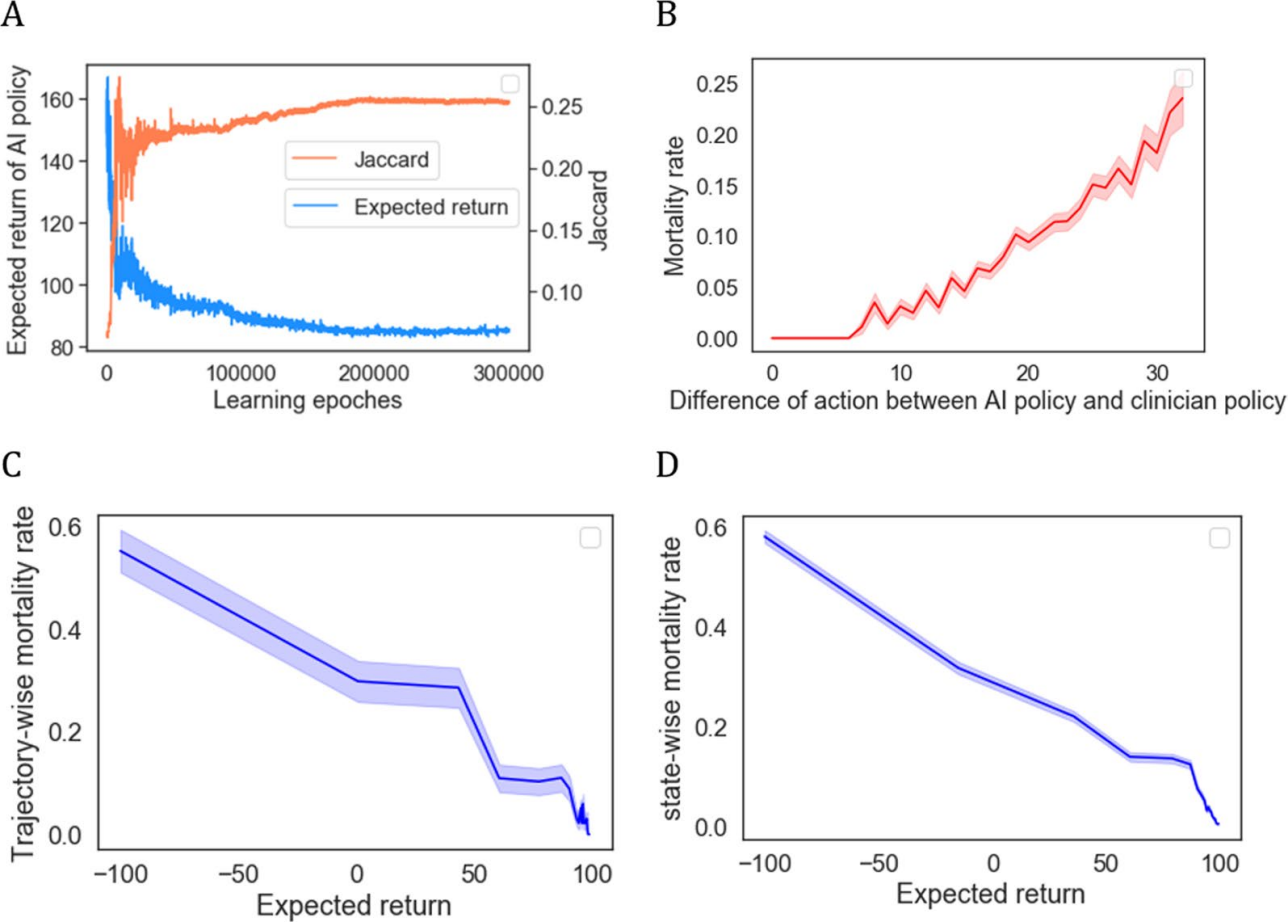
Model effectiveness and stability

Figure 4 further indicates the effectiveness and stability of the AI model. Figure 4A shows the expected return and Jaccard value obtained in each learning epoch, demonstrating that the AI model can maximize both the expected return and the similarity to the clinician policy and achieved stability after approximately 200,000 epochs. Figure 4B shows that the observed mortality rates varied with the difference in treatment actions between the AI policy and the clinician policy. The smallest treatment action difference was associated with the best survival rates. When the difference was not greater than 6, the in-hospital mortality rate was zero; the greater the discrepancy was in the clinicians prescribed drugs and those recommended by the AI model, the worse the outcome. Figure 4C, D shows the correlation between the expected returns of the clinicians’ treatment actions and the trajectory-wise and state-wise in-hospital mortality rates. We observed that treatment actions with low returns were associated with a high risk of mortalities, whereas treatments with high returns achieved better survival rates. This demonstrated that the estimated mortality calculating method could effectively reflect that in-hospital mortalities have clear negative correlation with the expected returns. Thus, the estimated mortalities generated according to the relationship between the distribution of the expected returns and the mortality rates were relatively reliable.

### Feature importance reflects the interpretability

**Fig. 5:**
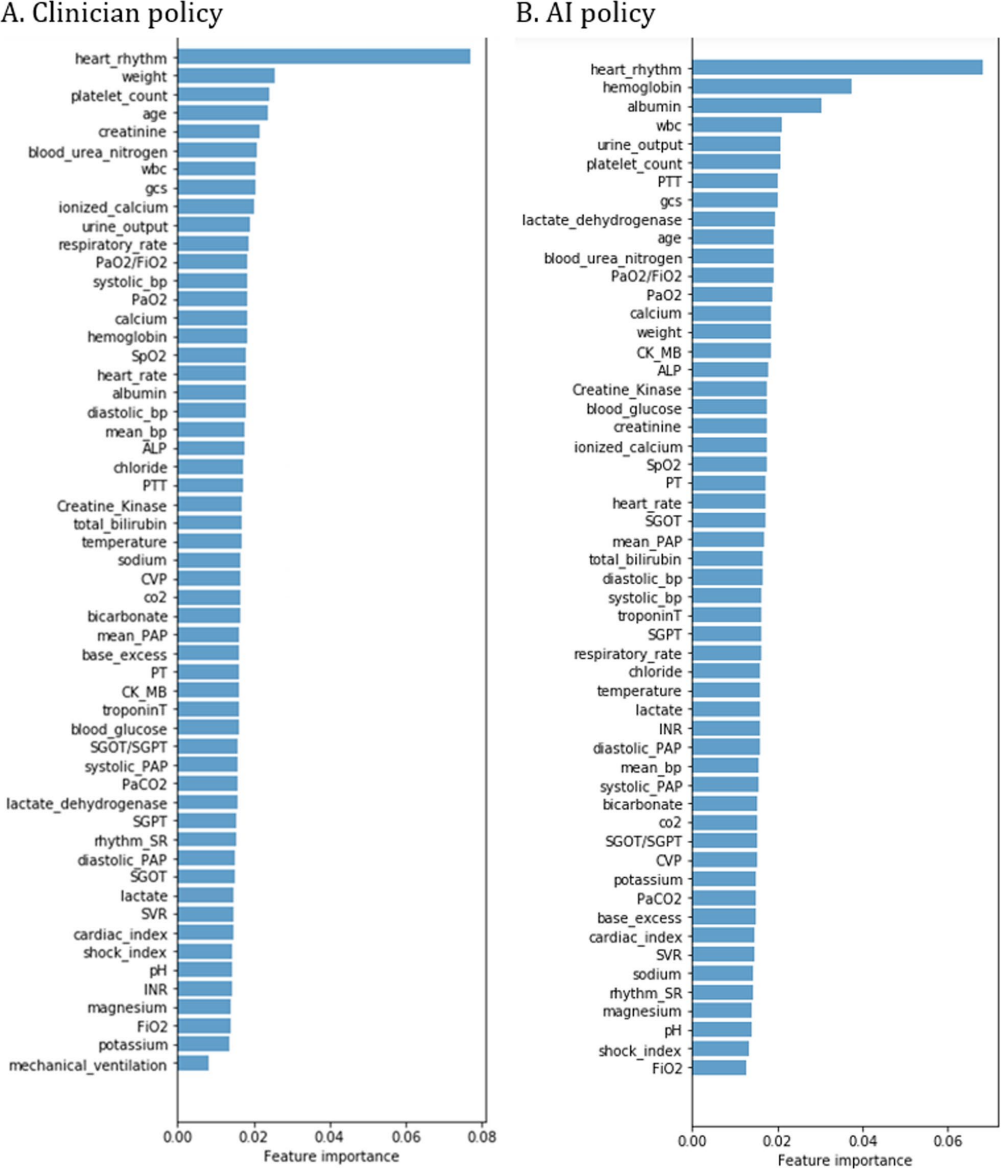
Feature importance in the clinician policy and the AI policy

Figure 5 shows the feature (except diagnosis) importance gained from the random forest model for the clinician policy and the AI policy generated by SRL-LSTM method respectively. These results confirmed that the treatment decisions made by both the clinician policy and the AI policy were clinically interpretable and relied primarily on sensible clinical and biological parameters. Among the ten most important features, both the clinician policy and the AI policy emphasized heart rhythm, WBC, urine output, platelet count, GCS score, and age; the clinician policy paid more attention to weight, creatinine, blood urea nitrogen, and ionized calcium; the AI policy was more concerned with haemoglobin, albumin, PTT, and lactate dehydrogenase.

### Comparison of the clinician and AI policies in case studies

**Fig. 6:**
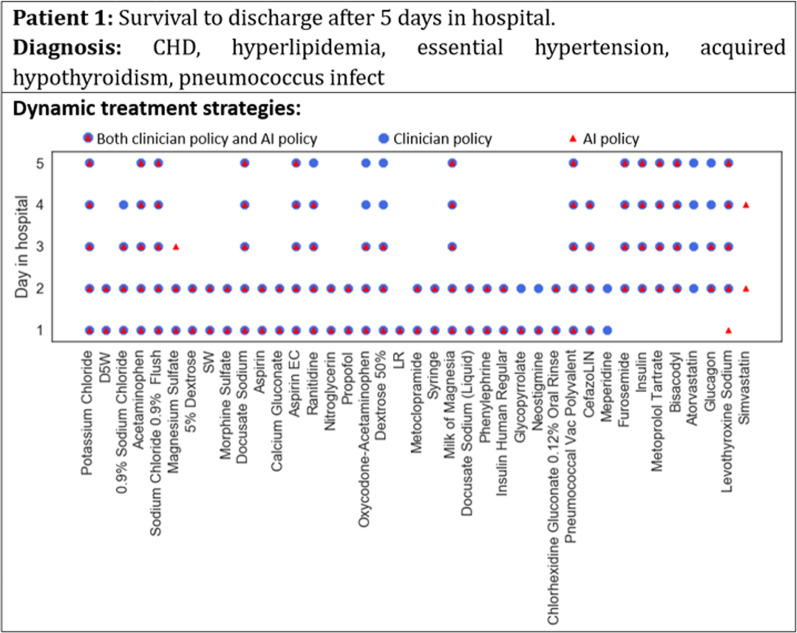
Case study of dynamic treatment strategies for a surviving patient

**Fig. 7:**
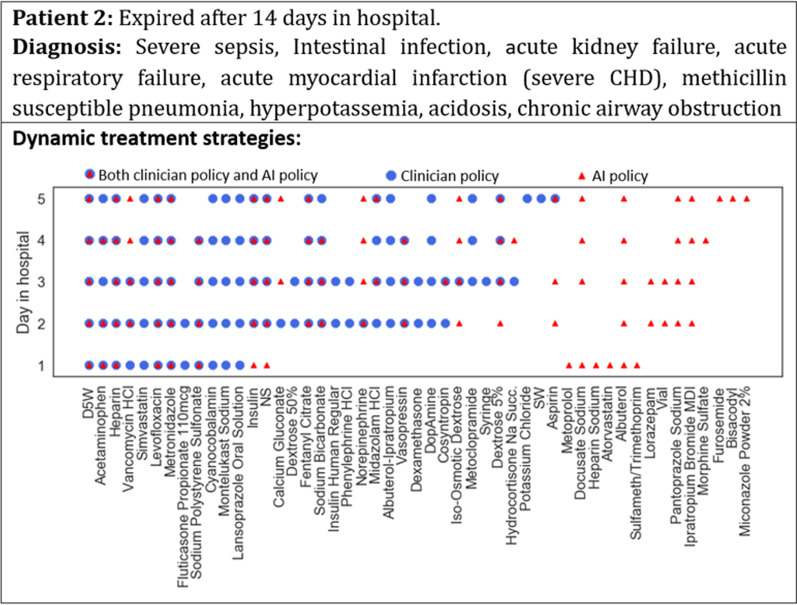
Case study of dynamic treatment strategies for an expired patient

Figures 6 and 7 show the dynamic treatment strategies generated by clinicians and AI for two patients on different hospital days. For patient 1 in Fig. 6, who survived to discharge after 5 days in the hospital, the similarity between the daily prescription of the clinician and AI policies was high, indicating that AI was able to learn the best practices of the clinicians. For patient 2 in Fig. 7, who expired after 14 days in the hospital, the daily prescription of the AI policy was quite different from that of the clinicians, and its rationality needs to be further examined by experts.

## Discussion

### Comparison with recent methods

The AI model of SRL-LSTM outperformed the recent methods in learning dynamic treatment strategies for CHD patients (Table 2). It could not only improve patient outcome, but also mimic the best practices of clinicians. The Dual-LSTM, AMANet and SL-LSTM were SL models with relatively high Jaccard values, indicating that they were capable of learning the experiences of the clinicians but had little ability to reduce the in-hospital mortality rate. The RL-LSTM model was supposed to be the most effective model for reducing the estimated in-hospital mortality rate. However, the Jaccard value was only 0.0342, one-tenth of the SL-LSTM model, and the average number of drugs recommended per patient per day was 230, ten times the average amount (23) clinicians prescribed. It was obviously not reasonable. DPO-LSTM was inferior to the SRL-LSTM model in both reducing the in-hospital mortality rate and mimicking the behaviours of the clinicians. SRL-Multimorbidity was inferior to the SRL-LSTM model for the CHD patients in both improving patient outcomes and mimicking the behaviours of the clinicians, indicating that the AI model developed for multimorbidity should not be directly used for a specific disease, such as CHD. It is essential to modify the models according to the characteristics and risk factors for the specific disease.

### Comparison with similar studies

The results of this study complied with the theoretical analysis and experimental results in similar studies in the literature. Many studies [12–16] have demonstrated that SL approaches are adept at learning the behaviours of doctors. RL approaches generate treatment recommendations by maximizing the expected return according to the reward mechanism, so they have the potential to recommend better treatment than those of the clinicians to improve patient outcomes [51]. For example, Weng [20] adopted an RL paradigm using policy iteration to learn the optimal glycaemic control policy for septic patients and found that the best optimal policy could potentially reduce the estimated mortality rate by 6.3%; Komorowski et al. [18] developed an AI clinician by using an RL approach of policy iteration to learn the optimal dynamic dosing of intravenous fluids and vasopressors for sepsis treatment. They found that the AI clinician recommended lower doses of intravenous fluids and higher doses of vasopressors than the clinicians’ actual treatments, the smallest dose difference was associated with the best survival rates, and the further away the dose received was from the suggested dose, the worse the outcome. Additionally, the SL approaches did not take the outcome of the patients into consideration when mimicking the practice of the clinicians; the RL approach, on the contrary, may recommend treatments that are obviously different from clinicians’ prescription due to the lack of supervision, which may be of high risk in the clinical practice [58]. These two approaches can complement each other. For example, Wang et al. [15] proposed a SRL framework for dynamic medication recommendations for multimorbidity, and the experiment on MIMIC-III illustrated that the SRL-multimorbidity model could reduce the estimated mortality, while providing promising accuracy in matching doctors’ prescriptions, which provided a prospect for combing SL and RL approaches.

### Limitations and future directions

This study was a preliminary exploration of learning dynamic treatment strategies for CHD patients, and more work is needed to make it practical. It is worthwhile to explore how to combine the structured data and the unstructured information (including the narrative diagnosis and other free-text records at bedside) to learn more practical dynamic treatment strategies. In addition, the AI model built in a pure data-driven way might be improved by leveraging domain knowledge of medicine and clinical guidelines to avoid major adverse drug-drug interactions. Moreover, this study focused only on whether to take specific drugs on each hospitalization day, and future studies need to further investigate the impact of drug doses. Further investigation is required to validate the effectiveness of the AI model in various CHD cohort and obtain careful evaluations from experts in medical domain.

This study aimed to learn optimal dynamic treatment strategies by using real-world data, thus was based on the following assumptions [32]: a. the consistency assumption that the state and results observed in each stage for each patient are such that potentially would be seen after the patient actually receives the corresponding treatment; b. the stable unit treatment value assumption that the potential outcome for each patient is not affected by treatments applied to other patients; c. the no unmeasured confounders, also referred to as the sequential randomization assumption. These assumptions were defaulted in this study, and further clinical trials or other prospective studies are needed to make the AI model practical.

Besides, despite AI technologies have the promise to support clinicians making more efficient and high quality treatment decisions, there are formidable obstacles and pitfalls, including risks for bias and overfitting, limited generalizability, risks of privacy and data security, and cause or exacerbate inequities [67–70]. Many initially promising technologies have failed in broader testing and applications. For example, Watson for Oncology that used by hundreds of hospitals worldwide for recommending treatments for cancer patients, provided many erroneous treatment recommendations, such as suggesting using bevacizumab in a patient with severe bleeding, which is an explicit contraindication [67, 71]. The Automated Retinal Disease Assessment (ARDA) tool was developed by Google to detect a condition that causes blindness in diabetic patients. Though ARDA was effective working with sample data, it struggled with images taken in field clinics during the test in a hospital in India [72]. The potential for an AI algorithm inducing iatrogenic risk is vast if it was widely applied. Therefore, when the AI algorithm is to be unleashed in clinical practice, systematic debugging, audit, extensive simulation and validation on various patient groups, along with prospective scrutiny and participation of different stakeholders, are required to ensure its efficiency and generalization [67–70]. Regulatory, governance and ethical guidelines are also necessary to ensure the information security, ethics and equity [67–70].

## Conclusion

We proposed a pipeline for constructing an AI model to learn dynamic treatment strategies for patients with CHD, the leading cause of death and one of the most serious epidemic diseases worldwide [1]. The cohort was selected following strict inclusion and exclusion criteria, and the features were extracted according to monitoring indexes and risk factors for CHD patients by referring to CHD-related guidelines [5–7], handbooks [4], reports [3], and papers [1, 2]. The AI model combining SL and RL resulted in better performance than using either SL or RL alone. The combined approach can help improve the outcomes of CHD patients and learn the best practices of clinicians and is clinically interpretable by relying on sensible clinical features. And a lot of further studies and efforts are needed to make it practical.

## Supplementary Information

**Additional file 1: Table S1.** Mean Euclidean distance of each group. **Table S2.** Hyperparameters adopted in the SRL-LSTM model. **Table S3.** Description of the CHD cohort.

**Additional file 2: Figure S1.** A visual example of the dynamic treatment process according to the diagnoses and time series variables of a CHD patient. A total of 50 drugs were prescribed during her 9 hospitalization days in the dynamic treatment strategy, and 10 were selected as an illustration.

**Additional file 3: Figure S2.** The framework of SRL-LSTM model.

## Data Availability

The datasets analysed during the current study were extracted from the MIMIC-III database, which is available in the PhysioNet repository, https://physionet.org/content/mimiciii/1.4/.

## Abbreviations

CHD: Coronary heart disease
ICU: Intensive care unit
EHRs: Electronic health records
AI: Artificial intelligence
SL: Supervised learning
AMANet: Attention and memory-augmented networks
RL: Reinforcement learning
LSTM: Long short-term memory
SRL: Supervised reinforcement learning
SRL-LSTM: SRL with an LSTM network
ICD: International Classification of Diseases
PAP: Pulmonary artery pressure
SVRI: Systemic vascular resistance index
GCS: Glasgow coma scale
CK: Creatine kinase
SGOT: Serum glutamic-oxaloacetic transaminase
SGPT: Serum glutamic pyruvic transaminase
PTT: Partial thromboplastin time
PT: Prothrombin time
INR: International normalized ratio
PaO2: Partial pressure of oxygen
CO2: Carbon dioxide
KNN: K-nearest neighbours
DDPG: Deep deterministic policy gradient
DPO: Direct policy optimization
ARDA: Automated Retinal Disease Assessment

## References

[1] Dai H, Much AA, Maor E (2020). Global, regional, and national burden of ischemic heart disease and its attributable risk factors, 1990–2017: results from the global Burden of Disease Study 2017. Eur Heart J Qual Care Clin Outcomes.

[2] Benjamin EJ, Muntner P, Alonso A (2019). Heart disease and stroke statistics—2019 update: a report from the American Heart Association. Circulation.

[3] National Center for Cardiovascular Diseases, China (2020). Annual report on cardiovascular health and diseases in China 2019.

[4] Zhu J (2019). Fuwai manual of cardiovascular critical care medicine.

[5] Knuuti J, Wijns W, Saraste A (2020). 2019 ESC guidelines for the diagnosis and management of chronic coronary syndromes. Eur Heart J.

[6] Joseph J, Velasco A, Hage FG (2018). Guidelines in review: comparison of ESC and ACC/AHA guidelines for the diagnosis and management of patients with stable coronary artery disease. J Nucl Cardiol.

[7] Committee of Experts on Rational Drug Use of National Health Commission of the P.R. China, Chinese Pharmacists Association (2018). Guidelines for rational drug use for coronary heart disease (Second edition). Chin J Front Med (Electron Ed).

[8] Zhao Y, Kosorok MR, Zeng D (2009). Reinforcement learning design for cancer clinical trials. Stat Med.

[9] Zhao Y, Zeng D, Socinski MA (2011). Reinforcement learning strategies for clinical trials in nonsmall cell lung cancer. Biometrics.

[10] Fang G, Annis IE, Elston-Lafata J (2019). Applying machine learning to predict real-world individual treatment effects: insights from a virtual patient cohort. J Am Med Inform Assoc.

[11] Sharma D, Aujla GS, Bajaj R (2019). Evolution from ancient medication to human-centered healthcare 4.0: a review on healthcare recommender systems. Int J Commun Syst.

[12] Fraccaro P, Castelerio MA, Ainsworth J (2015). Adoption of clinical decision support in multimorbidity: a systematic review. JMIR Med Inform.

[13] Bajor JM, Lasko TA. Predicting medications from diagnostic codes with recurrent neural networks. In: International conference on learning representations, April 24–26, 2017; Toulon, France.

[14] Zhang Y, Chen R, Tang J, et al. LEAP: learning to prescribe effective and safe treatment combinations for multimorbidity. In: Proceedings of the 23th ACM SIGKDD international conference on knowledge discovery & data mining 2017. pp. 1315–24. 10.1145/3097983.3098109.

[15] Wang L, Zhang W, He X, et al. Supervised reinforcement learning with recurrent neural network for dynamic treatment recommendation. In: Proceedings of the 24th ACM SIGKDD international conference on knowledge discovery & data mining 2018. pp. 2447–56. 10.1145/3219819.3219961.

[16] Gong F, Wang M, Wang H (2021). SMR: medical knowledge graph embedding for safe medicine recommendation. Big Data Res.

[17] Wang S. SeqMed: recommending medication combination with sequence generative adversarial nets. In: Proceedings of 2020 IEEE international conference on bioinformatics and biomedicine (BIBM); digital conference, pp. 2664–71. 10.1109/BIBM49941.2020.9313196.

[18] Komorowski M, Celi LA, Badawi O (2018). The artificial intelligence clinician learns optimal treatment strategies for sepsis in intensive care. Nat Med.

[19] Raghu A, Komorowski M, Ahmed I, et al. Deep reinforcement learning for sepsis treatment. In: 31st conference on neural information processing systems 2017, Long Beach, CA, USA.

[20] Weng W, Gao M, He Z, et al. Representation and reinforcement learning for personalized glycemic control in septic patients. In: 31st conference on neural information processing systems 2017, Long Beach, CA, USA.

[21] Bucur A, Leeuwen JV (2016). Workflow-driven clinical decision support for personalized oncology. BMC Med Inform Decis Mak.

[22] Jiang X, Wells A, Brufsky A (2019). A clinical decision support system learned from data to personalize treatment recommendations towards preventing breast cancer metastasis. PLoS ONE.

[23] Zhang B, Tsiatis AA, Laber EB (2013). Robust estimation of optimal dynamic treatment regimes for sequential treatment decisions. Biometrika.

[24] Zhu R, Zhao YQ, Chen G (2017). Greedy outcome weighted tree learning of optimal personalized treatment rules. Biometrics.

[25] Sun L, Liu C, Guo C, et al. Data-driven automatic treatment regimen development and recommendation. In: Proceedings of the 22nd ACM SIGKDD international conference on knowledge discovery & data mining 2016, pp. 1865–1874. 10.1145/2939672.2939866.

[26] Zheng H, Ryzhov IO, Xie W (2021). Personalized multimorbidity management for patients with type 2 diabetes using reinforcement learning of electronic health records. Drugs.

[27] Wang Y, Fu H, Zeng D (2018). Learning optimal personalized treatment rules in consideration of benefit and risk: with an application to treating type 2 diabetes patients with insulin therapies. J Am Stat Assoc.

[28] Ye X, Zeng QT, Facelli JC (2020). Predicting optimal hypertension treatment pathways using recurrent neural networks. Int J Med Inform.

[29] Zhang P, Wang F, Hu J, et al. Towards personalized medicine: leveraging patient similarity and drug similarity analytics. In: AMIA joint summits on translational science proceedings 2014, pp. 132–6. PMID: 25717413 PMC4333693 25717413

[30] Robins JM. Optimal structural nested models for optimal sequential decisions. In: Proceedings of the second Seattle symposium on biostatistics 2004, pp. 189–326. 10.1007/978-1-4419-9076-1_11.

[31] Gunlicks-Stoessel M, Mufson L, Westervelt A (2016). A pilot SMART for developing an adaptive treatment strategy for adolescent depression. J Clin Child Adolesc Psychol.

[32] Schulte PJ, Tsiatis AA, Laber EB (2014). Q- and A-Learning methods for estimating optimal dynamic treatment regimes. Stat Sci.

[33] Zhao Y, Zeng D, Rush AJ (2012). Estimating individualized treatment rules using outcome weighted learning. J Am Stat Assoc.

[34] Bremer V, Becker D, Kolovos S (2018). Predicting therapy success and costs for personalized treatment recommendations using baseline characteristics: data-driven analysis. J Med Internet Res.

[35] Zhang Y, Laber EB, Tsiatis A (2015). Using decision lists to construct interpretable and parsimonious treatment regimes. Biometrics.

[36] Zhang Y, Laber EB, Davidian M (2018). Interpretable dynamic treatment regimes. J Am Stat Assoc.

[37] Almirall D, Compton SN, Gunlicks-Stoessel M (2012). Designing a pilot sequential multiple assignment randomized trial for developing an adaptive treatment strategy. Stat Med.

[38] Shortreed SM, Laber E, Lizotte DJ (2011). Informing sequential clinical decision-making through reinforcement learning: an empirical study. Mach Learn.

[39] Liu Y, Logan B, Liu N (2017). Deep reinforcement learning for dynamic treatment regimes on medical registry data. Healthc Inform.

[40] Chen G, Zeng D, Kosorok MR (2016). Personalized dose finding using outcome weighted learning. J Am Stat Assoc.

[41] Zhou X, Mayerhamblett N, Khan U (2017). Residual weighted learning for estimating individualized treatment rules. J Am Stat Assoc.

[42] Huang Z, Ge Z, Dong W (2018). Probabilistic modeling personalized treatment pathways using electronic health records. J Biomed Inform.

[43] Huang Z, Lu X, Duan H (2012). On mining clinical pathway patterns from medical behaviors. Artif Intell Med.

[44] Chen Z, Marple K, Salazar E (2016). A physician advisory system for chronic heart failure management based on knowledge patterns. Theory Pract Logic Program.

[45] Chen Z, Salazar E, Marple K (2017). Improving adherence to heart failure management guidelines via abductive reasoning. Theory Pract Logic Program.

[46] Chen Z, Salazar E, Marple K (2018). An AI-based heart failure treatment adviser system. IEEE J Transl Eng Health Med.

[47] Hansen D, Dendale P, Coninx K (2017). The European association of preventive cardiology exercise prescription in everyday practice and rehabilitative training (EXPERT) tool: a digital training and decision support system for optimized exercise prescription in cardiovascular disease, concept, definitions and construction methodology. Eur J Prev Cardiol.

[48] Hauskrecht M. Dynamic decision making in stochastic partially observable medical domains: ischemic heart disease example. In: Keravnou E, Garbay C, Baud R, Wyatt J, editors. Artificial intelligence in medicine. AIME; 1997. 10.1007/BFb0029462.

[49] Krittanawong C, Johnson KW, Rosenson RS (2019). Deep learning for cardiovascular medicine: a practical primer. Eur Heart J.

[50] He Y, Wang C, Li N, et al. Attention and memory-augmented networks for dual-view sequential learning. In: Proceedings of The 26th ACM SIGKDD conference on knowledge discovery & data mining 2020. pp. 125–134. 10.1145/3394486.3403055

[51] Liu S, See KC, Ngiam KY (2020). Reinforcement learning for clinical decision support in critical care: a comprehensive review. J Med Internet Res.

[52] Chakraborty B, Murphy SA. Dynamic treatment regimes. In: Proceedings of the 32nd annual meeting of the society for medical decision making 2014. pp. 447–464. 10.1146/annurev-statistics-022513-115553.

[53] Boominathan S, Oberst M, Zhou H, et al. Treatment policy learning in multiobjective settings with fully observed outcomes. In: Proceedings of the 26th ACM SIGKDD conference on knowledge discovery & data mining 2020. pp. 1937–1947. 10.1145/3394486.3403245.

[54] Huang X, Goldberg Y, Xu J (2019). Multicategory individualized treatment regime using outcome weighted learning. Biometrics.

[55] Zhao YQ, Laber EB, Ning Y (2019). Efficient augmentation and relaxation learning for individualized treatment rules using observational data. J Mach Learn Res.

[56] Prescott HC, Sussman JB (2020). Smarter use of corticosteroids in treating patients with septic shock. JAMA Netw Open.

[57] Medicine IO (2006). To err is human: building a safer health system. Front Health Serv Manag.

[58] Mihatsch O, Neuneier R (2002). Risk-sensitive reinforcement learning. Mach Learn.

[59] Johnson A, Pollard T, Shen L (2016). MIMIC-III, a freely accessible critical care database. Sci Data.

[60] Johnson A, Pollard T, Mark R (2016). PhysioNet.

[61] Goldberger A, Amaral L, Glass L (2000). PhysioBank, PhysioToolkit, and PhysioNet: components of a new research resource for complex physiologic signals. Circulation.

[62] The British Heart Foundation. Coronary heart disease. https://www.bhf.org.uk/informationsupport/conditions/coronary-heart-disease. Accessed 28 May 2020.

[63] Free online searchable 2009 ICD-9-CM. http://icd9cm.chrisendres.com/index.php?action=contents. Accessed 28 May 2020.

[64] tf.keras.layers.Embedding. https://tensorflow.google.cn/api_docs/python/tf/keras/layers/Embedding. Accessed 26 July 2021.

[65] Sutton RS, Barto AG (2018). Reinforcement learning: an introduction.

[66] Lillicrap T, Hunt JJ, Pritzel A, et al. Continuous control with deep reinforcement learning. In: International conference on learning representations 2017. https://arxiv.org/pdf/1509.02971.pdf. Accessed 9 April 2020.

[67] Topol EJ (2019). High-performance medicine: the convergence of human and artificial intelligence. Nat Med.

[68] Tat E, Bhatt DL, Rabbat MG (2020). Addressing bias: artificial intelligence in cardiovascular medicine. Lancet Digit Health.

[69] Lopez-Jimenez F, Attia Z, Arruda-Olson AM (2020). Artificial intelligence in cardiology: present and future. Mayo Clin Proc.

[70] Ben AW, Pesaranghader A, Avram R (2021). Implementing machine learning in interventional cardiology: the benefits are worth the trouble. Front Cardiovasc Med.

[71] Ross C, Swetlitz I. IBM’s Watson supercomputer recommended ‘unsafe and incorrect’ cancer treatments, internal documents show. In Stat News. https://www.statnews.com/2018/07/25/ibm-watson-recommended-unsafe-incorrect-treatments/ (published 25 July 2018). Accessed 30 Dec 2021.

[72] Abrams C. Google’s effort to prevent blindness shows AI challenges. Dow Jones News. https://uk.advfn.com/stock-market/NASDAQ/GOOGL/share-news/Googles-Effort-to-Prevent-Blindness-Hits-Roadblock/79124560 (published 26 Jan 2019). Accessed 30 Dec 2021.

