# Supplementary material for: Learning dynamic treatment strategies for coronary heart diseases by artificial intelligence: real-world data-driven study
Source: BMC Med Inform Decis Mak. 2022 Feb 15;22:39. doi: 10.1186/s12911-022-01774-0 (PMC8845235; doi:10.1186/s12911-022-01774-0)
Supplement: Supplementary file 1 — Additional file 1: Table S1. Mean Euclidean distance of each group. Table S2. Hyperparameters adopted in the SRL-LSTM model. Table S3. Description of the CHD cohort. [file 12911_2022_1774_MOESM1_ESM.docx]

Supplementary materials for “**Learning Dynamic Treatment Strategies for Coronary Heart Diseases by Artificial Intelligence: Real-World Data-Driven Study.**”

Authors: Haihong Guo, Jiao Li, Hongyan Liu, Jun He

**Appendix Figure 1. A visual example of the dynamic treatment process according to the diagnoses and time series variables of a CHD patient.** A total of 50 drugs were prescribed during her 9 hospitalization days in the dynamic treatment strategy, and 10 were selected as an illustration.

**Appendix Table 1. Mean Euclidean distance of each group.**

| **Data set** | **Space** | **Closer group** | **Middle-distance group** | **Distant group** |
| --- | --- | --- | --- | --- |
| Development set | Original index space | 739.2045 | 1735.7176 | 3372.2900 |
|  | Embedding space | 0.0317 | 0.0426 | 0.0515 |
| Test set | Original index space | 747.1322 | 1752.4798 | 3436.1361 |
|  | Embedding space | 0.0304 | 0.0424 | 0.0512 |

**The framework and training process of SRL-LSTM model**

In SRL-LSTM model, the dynamic treatment strategy is modeled as a partially observed Markov decision process with finite time steps. For each patient admission $i$ in dataset ${D=\left\{ \left( S_{i,t}, A_{i,t}, {S_{i,t+1,} r}_{i,t} \right):t=1,\ldots,T_{i} \right\}}_{i=1}^{n}$, where *n* is the number of patient admission trajectories, $\left( S_{t}, A_{t}, S_{t+1}, r_{t} \right)$ is the transitions on the $t$^th^ day with the index $i$ omitted for simplicity, $S_{t}$ is the current observed state; $A_{t}$=($a_{t}^{1}$, $a_{t}^{2}$, $\ldots,a_{t}^{K}$) is the actual medications prescribed by clinicians, $K$ is the number of drugs and $a_{t}^{j}\in\left\{ 0,1 \right\}$ represents whether to take drug $j$; $S_{t+1}$ is the state on the next day after taken action $A_{t}$, and $S_{T_{i}+1}=0$ denotes the termination of the trajectory; $r_{t}$ is the reward gained. Given the state $S_{t}$, the SRL-LSTM model learns an optimal policy $\mu(S_{t}|\theta^{\mu})$ to select an action $\hat{A_{t}}$ that can maximize the expected return and learn to the clinicians’ experiences through a linear combination of RL and SL objectives. The input of the model is the current state, which is comprised of the embedded diagnoses, demographics and time-series variables of the patient till the current day, and the output is the action in the form of whether to take specific drugs predicted for the current state.

We used a deep deterministic policy gradient (DDPG) for the RL part, and cross entropy as the supervisor. In DDPG, there are two agents: actor $\mu(S|\theta^{\mu})$ and critic $Q(S,A|\theta^{Q})$, each of which has a target network $\mu^{'}(S|\theta^{\mu^{'}})$ and $Q^{'}(S,A|\theta^{Q^{'}})$with the same structure but different parameter update frequencies so as to ensure the robustness of the model. The network architect of actor $\mu(S|\theta^{\mu})$ is shown in Appendix Figure 2(a), which takes the state $S$ as input and outputs the predicted action, and the state is composed of diagnosed diseases, demographics and time-series variables. The network architect of critic $Q(S,A|\theta^{Q})$ is shown in Appendix Figure 2(b), which takes the state $S$ and action $A$ as input, and outputs their expected return, which metrics the quality of the action in this state. LSTM with a time window of 5 days was adopted to track the long term observed states within the actor and critic networks.

As shown in Appendix Figure 2(c), for a random mini-batch of $N$ transisions $\left( S_{t}, A_{t}, S_{t+1}, r_{t} \right)$ from data set $D$, the SRL-LSTM model was trained by the following steps:

Step 1, computing action probability predicted for the next state by the actor target network, i.e.${\hat{A_{t+1}}=\mu}^{'}\left( S_{t+1} | \theta^{\mu^{'}} \right)$, then let $\hat{A_{t+1}}\left[ \hat{A_{t+1}}>0.5 \right]=1$, $\hat{A_{t+1}}\left[ \hat{A_{t+1}}\leq0.5 \right]=0$. The input at this step is the next state $S_{t+1}$, which is comprised of the embedded diagnoses, demographics and time-series variables of the patient till the next day. The output at this step is the predicted action $\hat{A_{t+1}}$ on the next state;

Step 2, computing expected return for the next state by the critic target network, i.e. $Q^{'}(S_{t+1},\hat{A_{t+1}}|\theta^{Q^{'}})$, and then clipping it to $[-R_{max},R_{max}]$. The input at this step is the pair of next state $S_{t+1}$, and the next action $\hat{A_{t+1}}$ obtained at step 1, and the output is the expected return for the next state;

Step 3, computing the target expected return for the current state by $y = r_{t}+ \gamma Q^{'}(S_{t+1},\hat{A_{t+1}}|\theta^{Q^{'}})$. The inputs at this step are the real reward on the current state $r_{t}$ and the expected return for the next state $Q^{'}(S_{t+1},\hat{A_{t+1}}|\theta^{Q^{'}})$ obtained at step 2, and the output is the target expected return for the current state;

Step 4, computing the evaluated expected return for the current state by critic evaluation network, i.e. $Q\left( S_{t}, A_{t} | \theta^{Q} \right)$. The input at this step is the pair of the current state $S_{t}$ and the real action $A_{t}$ on the current state, where the current state $S_{t}$ is comprised of the embedded diagnoses, demographics and time-series variables of the patient till the current day. The output at this step is the evaluated expected return for the current state;

Step 5, computing the mean square error loss for critic evaluation network by $L_{Q}\left( \theta^{Q} \right)=\frac{1}{N} \sum_{i}^{N} {(y_{i}-Q\left( S_{i,t}, A_{i,t} | \theta^{Q} \right))}^{2}$. The inputs at this step are the target expected return for the current state $y$ obtained at step 3, and the evaluated expected return for the current state $Q\left( S_{t}, A_{t} | \theta^{Q} \right)$ obtained at step 4, and the output is the mean square error between the target and the evaluated expected return for the current state, which is the loss of the critic evaluation network;

Step 6, computing the gradient for critic evaluation network by GradientTape. The inputs at this step are the loss of the critic evaluation network obtained at step 5 and the trainable variables of the critic evaluation network, i.e. $\theta^{Q}$. The output at this step is the gradient for critic evaluation network;

Step 7, updating parameters $\theta^{Q}$ by 𝐴𝑑𝑎𝑚(𝑙𝑟𝑐) optimizer according to the gradient obtained at step 6;

Step 8, computing action probability predicted for current state by actor evaluation network, i.e. $\hat{A_{t}}= \mu\left( S_{t} | \theta^{\mu} \right)=\left( \hat{a_{t}^{1}}, \hat{a_{t}^{2}},\ldots,\hat{a_{t}^{K}} \right)$. The input in this step is the current state $S_{t}$, and the output is the action probability predicted for current state;

Step 9, computing the reinforcement learning loss of actor evaluation network, which is the negative mean of the expected return for the current state. So we first compute the expected return for the current state $Q\left( S_{t}, \hat{A_{t}} | \theta^{Q} \right)$ by the critic evaluation network, and then compute $L_{RL}\left( \theta^{\mu} \right)\approx-\frac{1}{N} \sum_{i} (Q\left( S_{t}, \hat{A_{t}} | \theta^{Q} \right))$. The input at this step is the pair of current state $S_{t}$ and the action probability $\hat{A_{t}}$ predicted for current state obtained at step 8. The output is the reinforcement learning loss of actor evaluation network, i.e. $L_{RL}\left( \theta^{\mu} \right)$;

Step 10, computing the supervised learning loss of actor evaluation network by $L_{SL}\left( \theta^{\mu} \right)=-\frac{1}{N} \sum_{i} \sum_{k=1}^{K} a_{t}^{k}\log(\hat{a_{t}^{k}})$. The inputs at this step are the real action on the current state and the action probability predicted for current state $\hat{A_{t}}$ obtained at step 8. The output at this step is the supervised learning loss of actor evaluation network, i.e. $L_{SL}\left( \theta^{\mu} \right)$;

Step 11, computing the combined loss of actor evaluation network by $L\left( \theta^{\mu} \right)= \varepsilon*L_{RL}\left( \theta^{\mu} \right)+\left( 1-\varepsilon\right)*L_{SL}\left( \theta^{\mu} \right)$. The inputs at this step are the reinforcement loss and the supervised loss of actor evaluation network obtained at step 9 and step 10 respectively, and the output is the combined loss of actor evaluation network;

Step 12, computing the gradient for actor evaluation network by GradientTape. The inputs at this step are the combined loss of actor evaluation network obtained at step 11, and the trainable variables of the actor evaluation network, i.e.$\theta^{\mu}$. The output at this step is the gradient for actor evaluation network;

Step 13, updating parameters $\theta^{\mu}$ by $Adam(lra)$ optimizer according to the gradient obtained at step 12;

Step 14, updating parameters of actor target network by $\theta^{\mu^{'}}= \tau\theta^{\mu}+\left( 1-\tau\right)\theta^{\mu^{'}}$, where $\theta^{\mu^{'}}$ is the trainable parameters of actor target network, and $\theta^{\mu}$ is the trainable parameters of actor evaluation network updated at step 13.

Step 15, updating parameters of critic target network by $\theta^{Q^{'}}= \tau\theta^{Q}+\left( 1-\tau\right)\theta^{Q^{'}}$, where $\theta^{Q^{'}}$ is the trainable parameters of critic target network, and $\theta^{Q}$ is the trainable parameters of critic evaluation network updated at step 7.

**Appendix Figure 2**. **The framework of SRL-LSTM model.**

The hyperparameters are summarized in Appendix Table 2. Parameters $di\_size$, $demo\_size$, $test\_size$, $feature\_size$, and $act\_size$ were set according to the variable size of the dataset. We tried two sets of values for the reward: one was 15/-15 for a patient discharged with life/died within hospital according to [15], and the other was 100/-100 according to [18], and the pre-experiment results showed that the later was more distinguished, so the later was chosen. $h_{0}$, the number of nodes for the hidden layer of diseases, was set to be 40, close to the $di\_size$; $h_{1}$, the number of nodes for the hidden layer of state and action, was set to be 500, equal to the $act\_size$. The time window for LSTM was set to be 5 according to [15]. We set the number of learning epochs as 200000 based on the pre-experiment, as the model achieved stability around 200,000 epochs. The other hyperparameters were set according to the common experience in computer sciences.

**Appendix Table 2. Hyperparameters adopted in the SRL-LSTM model.**

| **Parameters** | **Value** | **Description** |
| --- | --- | --- |
| $di\_size$ | 39 | There are at most 39 ICD-9 codes diagnosed for each admitted patient in the CHD cohort. |
| $demo\_size$ | 3 | 3 kinds of demographics included. |
| $test\_size$ | 76 | 52 time-series variables included, and among them the variable heart rhythm was divided into 25 one-hot coded sub-variables. |
| $feature\_size$ | 79 | Equal to $demo\_size$ plus test$\_size$ |
| $act\_size$*, K* | 500 | Number of drugs included, also the number of nodes on the output layer of the actor networks. |
| $time\_window$ | 5 | Number of states considered from the current state to the former states when making the action decision. |
| $r_{t}$ | 100,  -100,  0 | If the patient discharged with life, $r_{T}$ = 100; if the patient died within hospital, $r_{T}$ = -100; $r_{t}=0$ when $0<t<T,$ where $T$ is the total hospitalization days. |
| $R_{max}$ | 100 | A threshold for the expected return on the next state. |
| $\gamma$ | 0.99 | A discount factor to balance the importance of immediate and future rewards. |
| $h_{0}$ | 40 | Number of nodes for the hidden layer of diseases. |
| $h_{1}$ | 500 | Number of nodes for the hidden layer of state and action. |
| $\tau$ | 0.001 | The parameters update weight of the target network. |
| $lra$ | 0.001 | Learning rate of the actor evaluation network. |
| $lrc$ | 0.001 | Learning rate of the critic evaluation network. |
| $\varepsilon$ | [0,1] | The weight to trade off the objective between the supervised learning and the reinforcement learning. |
| $epoch\_count$ | 200000 | Number of learning epochs. |

**Appendix Table 3. Description of the CHD cohort.**

| **Items** | **Value** | **Items** | **Value** |
| --- | --- | --- | --- |
| Unique hospital admissions (N) | 13,762 | Unique patients (N) | 11,417 |
| Unique ICU admissions (N) | 14,795 | Drug included (N, %) | 500(98.0%) |
| Hospital characteristics | Teaching tertiary | Length of hospital stay, days (Median, IQR) | 9.2 (4.0 ~11.0) |
| Hospital location | Northeast of the USA | Length of ICU stay, days (Median, IQR) | 3.9 (1.24 ~ 4.11) |
| Unique hospitals (N) | 1 | **Procedure sites (N, %):** |  |
| Unique ICUs (N) | 5 | Cardiovascular | 10,575 (76.8%) |
| hospital mortality | 9.4% | Digestive system | 2,030 (14.8%) |
| **Source of hospital admission (N, %):** | | Respiratory system | 1,816 (13.2%) |
| Emergency department | 4338 (31.5%) | Nervous system | 1,587 (11.5%) |
| Transfer from external hospital | 3763 (27.3%) | Endocrine system | 1,202 (8.7%) |
| Physician referral | 2880 (20.9%) | Musculoskeletal system | 628 (4.6%) |
| Clinic referral | 2712 (19.7%) | Integumentary system | 423 (3.1%) |
| Other | 69 (0.5%) | Urinary system | 195 (1.4%) |
| **Type of first ICUs (N, %):** |  | Nose, mouth, pharynx | 159 (1.2%) |
| CCU/CSRU | 8983 (60.7%) | Hemic and lymphatic | 153 (1.1%) |
| MICU | 3820 (25.8%) | Eye | 104 (0.8%) |
| SICU/TSICU | 1992 (13.5%) | Other sites | 9450 (68.7%) |
| **Primary ICD-9 diagnosis (N, %):** | | **Other accompanying diseases (N, %):** | |
| CHD | 5,626 (40.9%) | Other circulatory system diseases | 13,015 (94.6%) |
| Other circulatory diseases | 3,131 (22.8%) |  |  |
| Injury and poisoning | 1,059 (7.7%) | Endocrine, Nutritional, Metabolic, Immunity diseases | 11,110 (80.7%) |
| Respiratory system diseases | 861 (6.3%) |  |  |
| Digestive system diseases | 858 (6.2%) | Genitourinary system diseases | 6,410 (46.6%) |
| Neoplasms | 439 (3.2%) |  |  |
| Infectious and parasitic diseases | 246 (1.8%) | Respiratory system diseases | 6,242 (45.4%) |
| Endocrine, Nutritional, Metabolic, Immunity diseases | 230 (1.7%) | Infectious and parasitic diseases | 5,676 (41.2%) |
| Genitourinary system diseases | 201 (1.5%) | Injury and poisoning | 5,526 (40.2%) |
| Musculoskeletal and connective tissue diseases | 109 (0.8%) | Digestive system diseases | 5,274 (38.3%) |
| Nervous system and sense organs diseases | 96 (0.7%) | Blood and blood forming organs diseases | 4,995 (36.3%) |
| Congenital anomalies | 41 (0.3%) | Nervous system and sense organs diseases | 3,871 (28.1%) |
| Blood and blood forming organs diseases | 33 (0.2%) |  |  |
|  |  | Mental disorders | 3,633 (26.4%) |
| Mental disorders | 20 (0.1%) | Musculoskeletal and connective tissue diseases | 2,640 (19.2%) |
| Other | 108 (0.8%) |  |  |
|  |  | Neoplasms | 1,519 (11.0%) |
|  |  | Congenital anomalies | 409 (3.0%) |
|  |  | Other | 5,398 (39.2%) |

Notes: CCU: Coronary Care Unit; CHD: Coronary heart disease; CSRU: Cardiac Surgery Recovery Unit; ICD-9: International Classification of Diseases version 9; ICU: Intensive Care Unit; IQR: Interquartile Range; MICU: Medical Intensive Care Unit; OASIS: Oxford Acute Severity of Illness Score; SD: Standard Deviation; SICU: Surgical ICU; TSICU: Trauma/surgical ICU.
