# Supplementary material for: Learning dynamic treatment strategies for coronary heart diseases by artificial intelligence: real-world data-driven study
Source: BMC Med Inform Decis Mak. 2022 Feb 15;22:39. doi: 10.1186/s12911-022-01774-0 (PMC8845235; doi:10.1186/s12911-022-01774-0)
Supplement: Supplementary file 2 — Additional file 2: Figure S1. A visual example of the dynamic treatment process according to the diagnoses and time series variables of a CHD patient. A total of 50 drugs were prescribed during her 9 hospitalization days in the dynamic treatment strategy, and 10 were selected as an illustration. [file 12911_2022_1774_MOESM2_ESM.pdf]

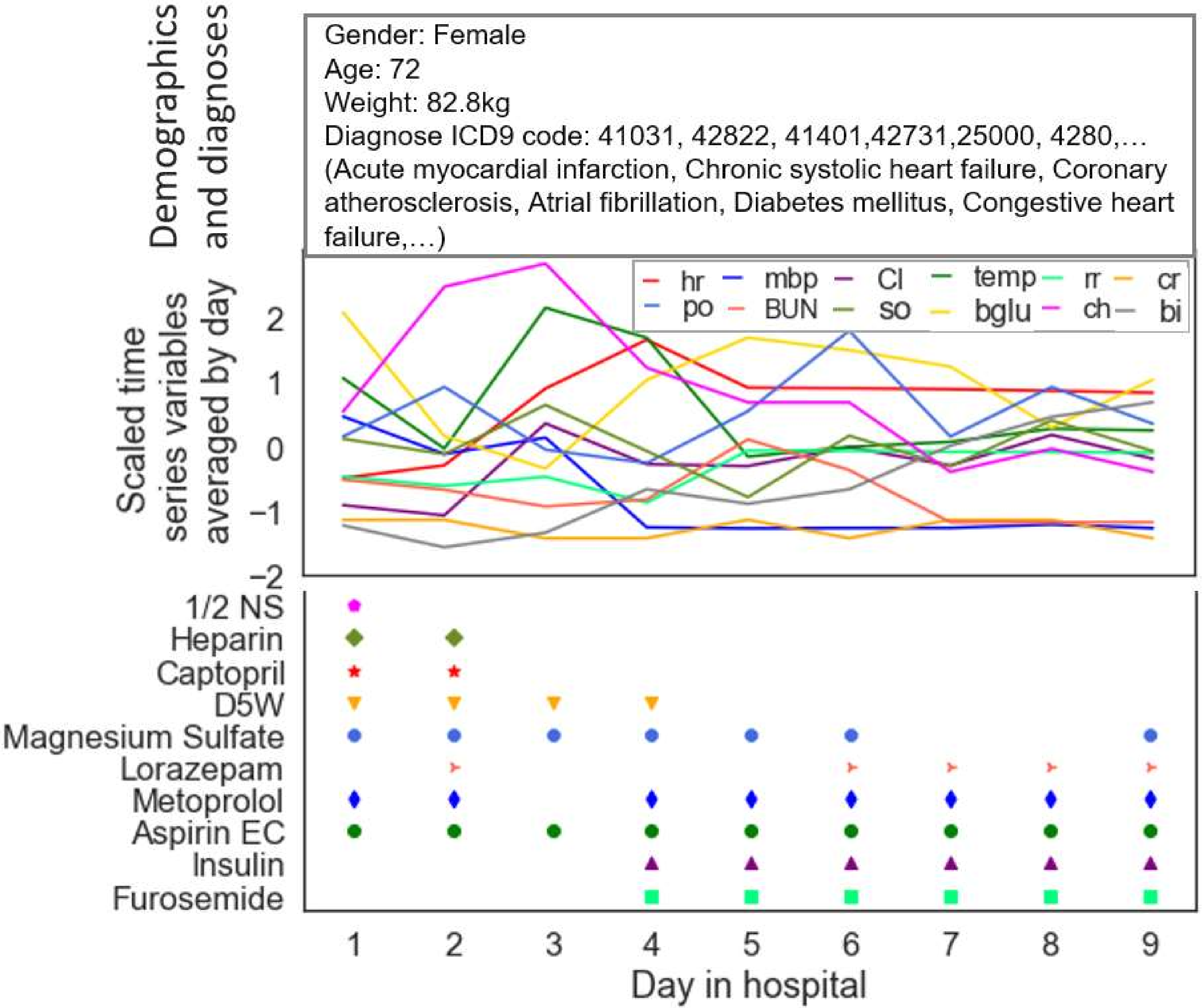

Demographics and diagnoses
Gender: Female
Age: 72
Weight: 82.8kg
Diagnose ICD9 code: 41031, 42822, 41401,42731,25000, 4280,…
(Acute myocardial infarction, Chronic systolic heart failure, Coronary atherosclerosis, Atrial fibrillation, Diabetes mellitus, Congestive heart failure,…)
Scaled time series variables averaged by day
hr
mbp
Cl
temp
rr
cr
po
BUN
so
bglu
ch
bi
2
1
0
−1
−2
1/2 NS
Heparin
Captopril
D5W
Magnesium Sulfate
Lorazepam
Metoprolol
Aspirin EC
Insulin
Furosemide
1
2
3
4
5
6
7
8
9
Day in hospital
