# Supplementary material for: Learning dynamic treatment strategies for coronary heart diseases by artificial intelligence: real-world data-driven study
Source: BMC Med Inform Decis Mak. 2022 Feb 15;22:39. doi: 10.1186/s12911-022-01774-0 (PMC8845235; doi:10.1186/s12911-022-01774-0)

A. Actor network $\mu(S|\theta^{\mu})$ and $\mu'(S|\theta^{\mu'})$

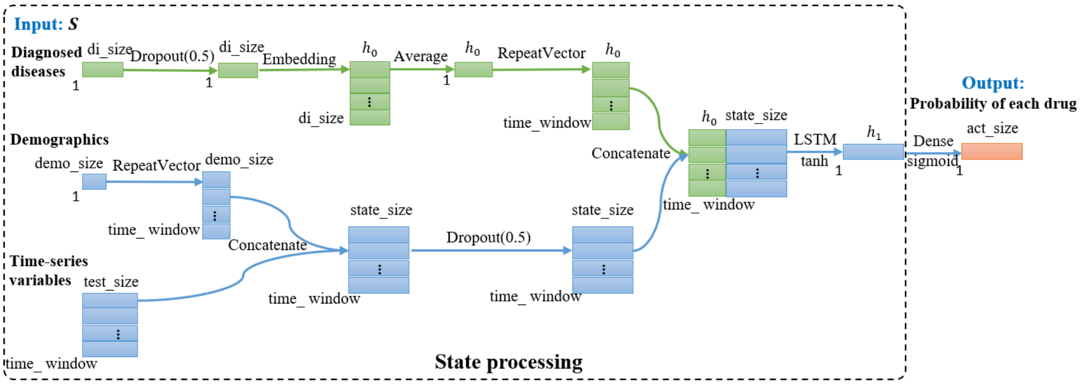

B. Critic network $Q(S,A|\theta^{Q})$ and $Q'(S,A|\theta^{Q'})$

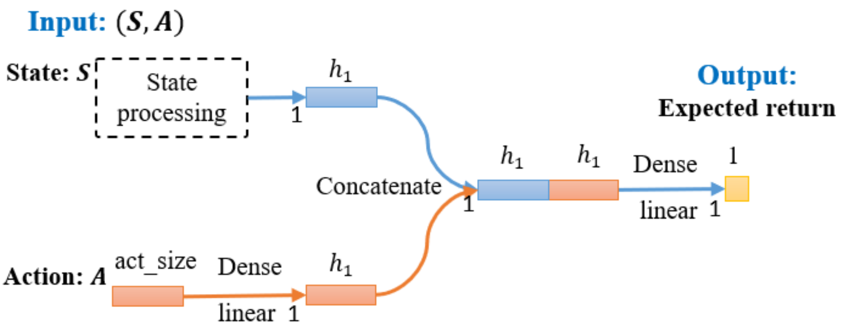

C. Training process of the SRL-LSTM model

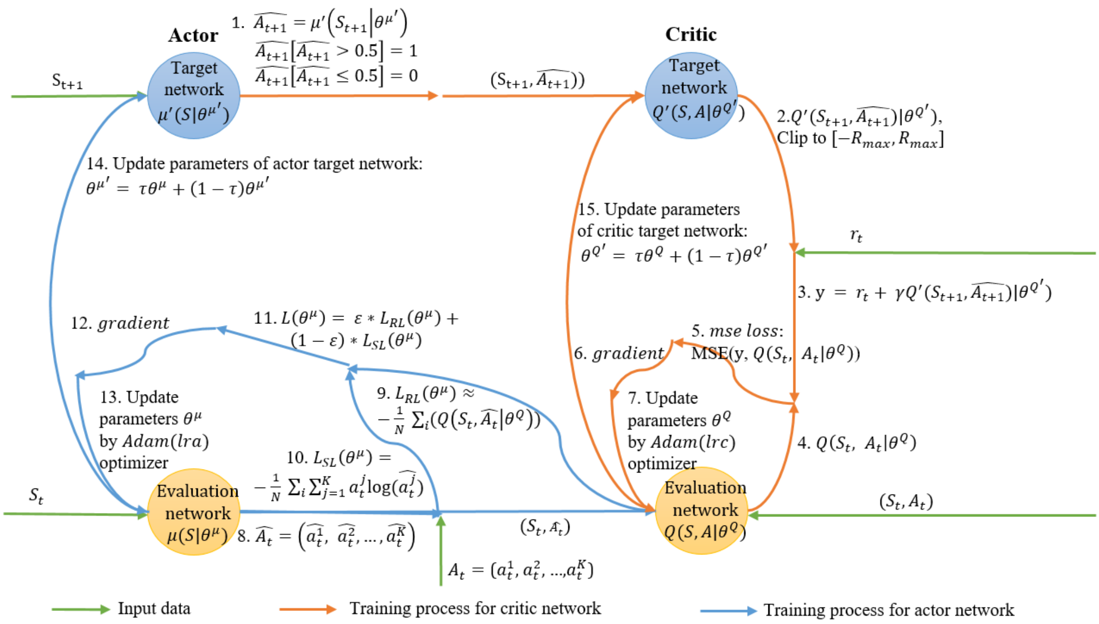

Supplement: Supplementary file 3 — Additional file 3: Figure S2. The framework of SRL-LSTM model. [file 12911_2022_1774_MOESM3_ESM.pdf]
